# *BRAF* Mutation in Colorectal Rhabdoid and Poorly Differentiated Medullary Carcinomas

**DOI:** 10.3390/cancers11091252

**Published:** 2019-08-26

**Authors:** Elena Bolzacchini, Nunzio Digiacomo, Cristina Marrazzo, Nora Sahnane, Roberta Maragliano, Anthony Gill, Luca Albarello, Fausto Sessa, Daniela Furlan, Carlo Capella

**Affiliations:** 1 Unit of Oncology, ASST-Lariana, 22100 Como, Italy; 2 Unit of Pathology, Department of Medicine and Surgery and Research Center for the Study of Hereditary and Familial tumors, University of Insubria, 21100 Varese, Italy; 3 Unit of Oncology, ASST-Sette Laghi, 21100 Varese, Italy; 4 Royal North Shore Hospital St Leonards, Cancer Diagnosis and Pathology Group, Kolling Institute of Medical Research, University of Sydney, Sydney NSW 2006, Australia; 5 Unit of Pathology, Ospedale San Raffaele, 20100 Milan, Italy

**Keywords:** *BRAF*, colorectal rhabdoid carcinomas, microsatellite instability, CpG island methylator phenotype, *SMARCB1*

## Abstract

Colorectal rhabdoid carcinomas (CRbCs) are very rare and aggressive cancers. The *BRAF* mutation and CpG island methylator phenotype have been reported to be common features of CRbCs. This study reviews the literature about CRbCs and analyzes the clinicopathological and molecular profiles of seven CRbCs characterized by large discohesive cells with abundant eosinophilic cytoplasm, showing hyaline inclusions and large rounded to bean-shaped nuclei. For comparison, we included four poorly differentiated medullary carcinomas (PDMCs) with focal aspects mimicking rhabdoid features. Overall survival was poor in both subsets, with 78% of patients dying of disease within 2–11 months. The main features of CRbCs were: Loss of/reduced SMARCB1/INI expression, intense vimentin immunostaining, and dense neutrophilic infiltration. The PDMCs were positive for pancytokeratin but negative for vimentin and showed moderate peritumoral/intratumoral CD8+ lymphocytes. All PDMCs showed SMARCB1(INI-1) expression. The coexistence of *BRAF* and *TP53* mutations was observed in 80% of CRbCs and PDMCs. PDMCs always showed microsatellite instability and CpG island methylator phenotype (CIMP), while CRbCs were CIMP negative and exhibited microsatellite instability (MSI) in two out of seven cases. CRbCs are characterized by *BRAF* and *TP53* mutations. Loss/reduced expression of nuclear SMARCB1/INI, intense vimentin immunostaining, dense neutrophilic infiltration, and low frequency of CIMP are useful markers to recognize these rare aggressive tumors.

## 1. Introduction

Colorectal rhabdoid carcinomas (CRbCs) are rare neoplasms, most often localized to the proximal colon in elderly patients with a mean age at diagnosis around 70 years. The prognosis of affected patients is poor due to the aggressive behavior of this disease, characterized by an overall survival shorter than 12 months. Only 39 cases of CRbCs have been reported in the literature as far as we know [1]. The most noteworthy histological feature of CRbCs is the rhabdoid cell containing eosinophilic aggregates of intermediate filaments that displace the nucleus to the cell periphery. Many cases have a pure rhabdoid morphology although they can be combined with more conventional forms of colorectal carcinomas (CRC) suggesting that they should be interpreted as rhabdoid carcinomas and not rhabdoid neoplasm equivalents of pediatric malignant rhabdoid tumors of the kidney and of the soft parts characterized by genetic inactivation of *SMARCB1* (SNF5, INI-1), a component of the switching/sucrose nonfermenting (SWI/SNF) chromatin remodeling complex [2].

The events involved in CRbC pathogenesis remain poorly elucidated and include *SMARCB1* (INI-1) inactivation [1,2], *BRAF* V600E mutation [1,3,4], epigenetics events and molecular features occurring in the serrated pathway [3], and inactivation of centrosomal functions due to *CROCC* mutation [4] (Table 1).

We herein report seven new cases of this entity, examining in detail their clinicopathologic features. For comparison, we included four poorly differentiated medullary carcinomas (PDMCs) with focal aspects mimicking rhabdoid features. Immunohistochemical, genetic, and epigenetic analyses were performed to clarify the molecular alterations associated with this phenotype, with special emphasis on *BRAF* V600E mutation. In addition, we reviewed the literature on the cases of this entity reported so far.

## 2. Results

### 2.1. Clinicopathological Features

The main clinicopathologic features of the seven patients with CRbC are reported in Table 2. Patients were four males and three females, aged 63–85 years (mean age: 70.5 years; median age: 65 years). Four tumors were localized in the right colon (one in the cecum, one in the ascending colon, and two in the hepatic flexure) and three in the left colon (one in the splenic flexure, one in the sigmoid colon, and one in the rectum). Presenting symptoms included nonspecific abdominal symptoms, weight loss, and evidence of gastrointestinal bleeding. All patients underwent radical surgical procedures (right-sided or left-sided colectomy with node dissection). Five of the six patients, for whom detailed data were available, had positive regional nodes and three of them also had intra-abdominal and/or liver metastases. The stage evaluated after surgical procedures was IIA for one patient, IIIB for two patients, IIIC for two patients, IVA for one patient, and was not defined in one case. Five patients died of disease within 2–11 months (mean 5.5 months) after surgery. Two patients are still alive (May 2019) 186 months and 216 months, respectively, after surgery.

Patients with PDMCs were two males and two females aged 53–94 years (mean: 75 years; median 76 years). Three neoplasms were in the right colon (two in the ascending colon and one in the cecum) and one neoplasm was in the left colon (sigmoid colon). All four patients underwent radical surgical treatment, and all were at stage IIIC. Two patients died of disease within 5–11 months (average 8 months), one patient is still alive after 124 months (May 2019). For one case, the follow-up was not available.

### 2.2. Pathologic Findings

**Figure 1:**
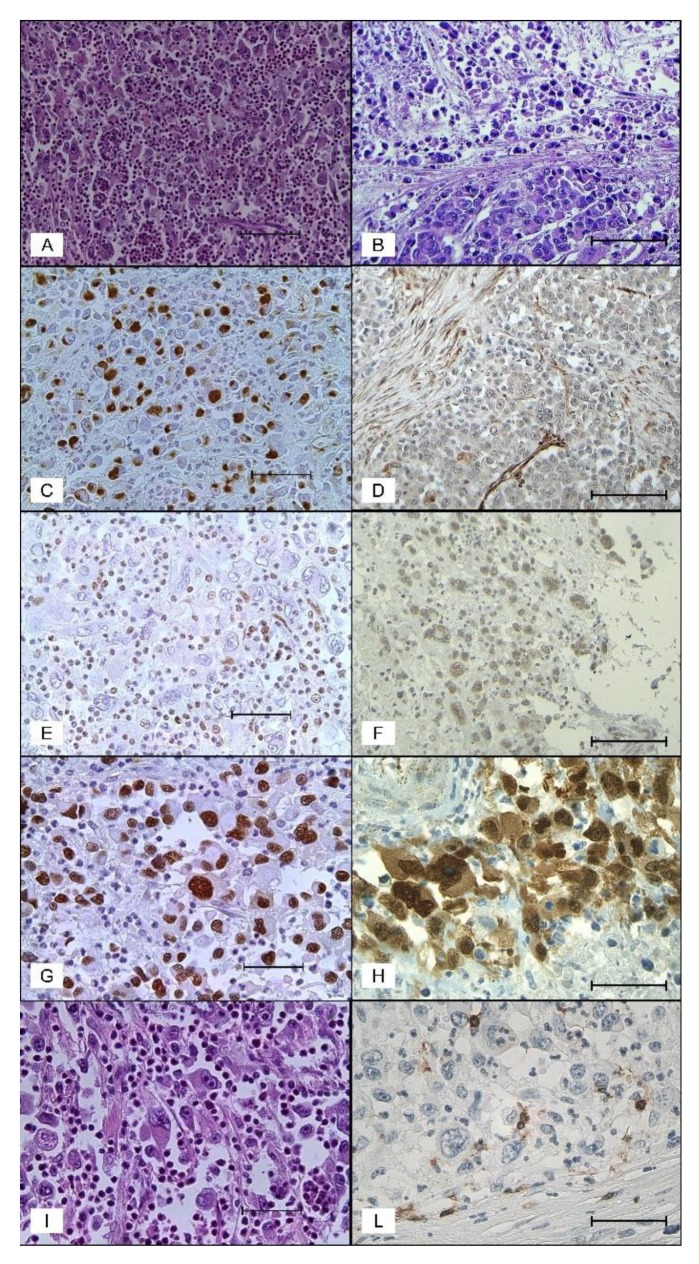
Morphological and immunohistochemical features in CRbCs and PDMCs. (**A**) and (**I**) CRbC showing non-cohesive rhabdoid cells admixed with numerous neutrophils (hematoxylin and eosin stain, 200× and 400×, scale bar 100 and 50 µm); (**B**) PDMC showing a cohesive medullary area adjoining an area of loosely cohesive cells (hematoxylin and eosin stain, 200×, scale bar 100 µm); (**C**) CrbC with strong immunostaining for vimentin predominantly in the paranuclear region of the cytoplasm (vimentin, 200×, scale bar 100 µm); (**D**) PDMC showing negative immunostaining for vimentin (vimentin, 200×, scale bar 100 µm); (**E**) complete loss of SMARCB1 (INI) expression) in a CRbC (INI-1, 400×, scale bar 50 µm); (**F**) loosely cohesive area of a PDMC showing SMARCB1 (INI) nuclear positivity (INI-1, 200×, scale bar 100 µm); (**G**) p53 nuclear expression in a CRbC (p53, 400×, scale bar 50 µm); (**H**) beta catenin nuclear expression in a CRbC (beta catenin, 400×, scale bar 50 µm); (**L**) CRbC showing few CD8-positive tumor infiltrating lymphocytes (CD8, 400×, scale bar 50 µm).

Grossly, the neoplasms (both CRbCs and PDMCs) were reported as huge ulcerated masses completely replacing the intestinal wall, measuring from 4–13 cm (average size: 7.6 cm). Histologically, CRbCs consisted of sheets of poorly cohesive cells subdivided in clusters by delicate strands of stroma (Figure 1A). Two types of neoplastic cells were found: Large pleomorphic rhabdoid cells and smaller round to polygonal cells. The large cells had pleomorphic eccentrically located nuclei and abundant cytoplasm containing large eosinophilic paranuclear “rhabdoid” inclusions (Figure 1A,I). The smaller cells were more uniform in size and had less abundant cytoplasm with less evident eosinophilic bodies. The proportion of the different cell types varied between tumors. The rhabdoid cellular aspects were predominant (>75%) in five cases and patchy in two cases. One case had a focal adenocarcinomatous component and another case showed solid medullary areas that blended with rhabdoid areas. Extensive coagulative necrosis was detected in all cases. All neoplasms contained among the tumor cells many infiltrating neutrophils with several cells showing emperipolesis. Tumor budding was present in all cases and graded as high. On the contrary, both the intratumoral and peritumoral lymphoid infiltration was absent or inconspicuous in all cases. Prominent vascular space invasion was detected in all cases and perineural invasions in two cases. Mitoses per 2 mm^2^ varied from 8–38 (average 16).

PDMCs contained solid areas of medullary carcinoma alternating with areas of loosely cohesive medium to large sized cells with eccentric nuclei and eosinophilic cytoplasm, but without well-defined paranuclear cytoplasmic hyaline inclusions (Figure 1B). Focal areas of glandular differentiation with mucin production and groups of signet ring cells were identifiable in two cases. Compared to CRbCs, PDMCs showed more frequent expansive growth, minor budding, more abundant stromal component, more consistent peritumoral lymphoid infiltration, and comparable tumor necrosis and vascular spaces invasion.

### 2.3. Immunohistochemical Findings

Immunohistochemistry of CRbCs showed strong cytoplasmic paranuclear positivity for vimentin in the majority (≥75%) of tumor cells (Figure 1C) in five cases and in significant areas ((≥30%) of two cases (Table 3). Pancytokeratin was variously positive in all cases. Six of the seven cases were positive for epithelial membrane antigen (EMA). Complete loss or reduced expression of nuclear SMARCB1 (INI-1) (Figure 1E) was found in five and two neoplasms, respectively. β-catenin displayed a variable (20%–100%) nuclear staining (Figure 1H) in five out of seven cases. p53 (Figure 1G) was strongly expressed (≥60% of neoplastic cells) in all cases. The proliferative index (Ki-67) varied from 38–90% (average 58%). The average number of CD8+ peritumoral lymphocytes was 31.7 (range 5–73) and that of intratumoral CD8+ lymphocytes 17.1 (range 3–38) (Figure 1L). All the remaining immunohistochemical markers including CK7, CK20, CDX-2, synaptophysin, and desmin were negative.

The four PDMCs were positive for pancytokeratin, but negative for vimentin (Figure 1D). All cases showed intact nuclear SMARCB1 (INI-1) expression. Nuclear β-catenin positive staining (in 15–80% of tumor cells) was present in two of four cases. TP53 positivity (in ≥60% cells) was observed in two of four neoplasms. The proliferation index (Ki-67) varied from 60–80% (mean 70%). The mean number of CD8+ peritumoral lymphocytes was 52 (range 23–106) and that of intratumoral CD8+ lymphocytes was 6.5 (range 2–15) CK7 and CK20 were negative in all cases. CDX2 immunoreactivity was found in two of four cases.

### 2.4. Molecular Findings

MSI was observed in two out of seven (28%) CRbCs and in all four PDMCs (Table 4). CIMP analysis was possible in a total of nine neoplasms including six CRbCs and three PDMCs. CIMP-H was observed only in the three PDMCs while all the CRbCs were classified as CIMP-negative. Raw data obtained for CIMP analysis are reported in Table S1. *MLH1* was methylated in all four MSI cancers in which the CIMP status was assessed.

Somatic mutation analysis by next gereration sequencing (NGS) sequencing was possible in all cases except for the CRbC 7 sample, showing high levels of DNA fragmentation and degradation. NGS analysis showed an average of 1,306,777 reads per sample with a median read length of 119 bp. The mean number of mapped reads in targeted regions per sample was 183,856, and average coverage per sample was 1689. *BRAF* and *TP53* mutations were observed in almost all tumors, occurring in nine out of 10 cases (five CRbCs and four PDMCs) and in nine out of 10 neoplasms (six CRbCs and three PDMCs), respectively. *BRAF* V600E mutation was detected in all cases except for CRbC 3 exhibiting a coexistence of NRAS G12D with a class III *BRAF* variant (i.e., *BRAF* G466A). *TP53* mutations were mainly missense pathogenetic variants in 273, 245, 272, and 278 codons, while only two *TP53* frameshifts mutations were observed in one case (PDMC 1). Two *KRAS* mutations were found in two *BRAF* wild-type cases, whereas *PIK3CA* mutations were detected in one CRbC and in one PDMC (Table 4). Raw data obtained for NGS analysis using CLC Genomics Workbench software are reported in Table S2.

For most cases exhibiting co-occurrence of *BRAF* and *TP53* mutations, *TP53* mutant allelic fractions (mAFs) were higher than *BRAF* mAFs (Table 3). Interestingly, in three cases showing *BRAF* mAFs higher than *TP53* mAFs, we found two simultaneous *TP53* mutations (case PDMC 1) or coexistence of *TP53* and *PIK3CA* mutations (cases CRbC 6 and PDMC 3). These findings are consistent with the “two-hits hypothesis” for tumor-suppressor genes but also suggest a driver role of anti-apoptotic/pro-survival pathways in these tumors that may be likely involved very early and together with the constitutive activation of the mitogen-activated protein kinase (MAP) kinase pathway.

In two cases, we observed *KRAS* or *BRAF* mAFs >50% (CRbC 5 and PDMC 3) that were suggestive of copy number gains in wild-type alleles.

### 2.5. Literature Review

A careful review of the MEDLINE literature revealed 39 cases of CRbCs that should be added to our seven cases for a total of 46 cases [1,2,4,5,6,7,8,9,10,11,12,13,14,15,16,17,18,19,20,21,22,23,24,25,26] (Table S3). The patients were 22 males and 24 females aged 23–87 years (mean age: 64 years; median 69 years). The neoplasms were located in order of frequency in the cecum (26%), sigmoid colon (17.4%), ascending colon and rectum (13% each), right colon unspecified (10.8%), transverse colon (8.7%), hepatic flexure, splenic flexure, and left colon unspecified (2.1% each). The size of the tumors was reported in 33 cases and ranged from 3–15 cm in maximum diameter (mean 7.3 cm, median 7 cm). Positive regional lymph nodes were found in 32/39 (82%) in which detailed data were available. Liver metastases were reported in 31% of the patients and peritoneal metastases in 10% of the cases.

Of the 36 patients with available data, 34 received surgery as first or unique treatment and three (8%) of them received adjuvant therapy. The remaining patients underwent palliative treatment because of advanced disease. Fluoropyrimidine- and oxaliplatin-based chemotherapy were the regimens mostly used as first line therapy in a metastatic setting. Other chemotherapy regimens used comprehended irinotecan (FOLFIRI scheme) [6] and anthracycline/platinum (EOX scheme) [9]. Only two patients [6,12] received more than one line of chemotherapy in a metastatic setting and only one patient received monoclonal antibody (both antivascular endothelial growth factor (anti-VEGF) and anti–epidermal growth factor (anti-EGFR)) with no benefit [12]. Follow-up data were available for 41 patients: 31 (75%) died of disease within 1–15 months (mean: 4.5 months, median 4 months). Ten patients with follow-up ≥6 months (range 6–216 months, mean 59 months, median 29.5 months) were reported as still alive.

In the histopathological reports, the rhabdoid cells were the prevalent cells in the majority of cases; however, in 45% of 35 cases an adenocarcinomatous component more frequently focal and at the tumor periphery and often poorly differentiated has been detected. The reported immunohistochemical findings proved in 36/36 cases coexpression of vimentin and pancytokeratin mainly localized to paranuclear cytoplasmic inclusions. Variable positivity for EMA has been found in 15/21 (71.4%) cases. Complete loss or focal loss of SMARCB1 (INI-1) was reported in 9/27 (33.5%) and 3/27 (11.1%) cases, respectively. Nuclear immunoreactivity for TP53 and β-catenin has been found in 12/34 (35.6%) and six of eight (75%) cases, respectively.

E-cadherin immunoreactivity was negative in the four cases investigated. CK20 and CDX2 were not expressed in the majority of cases: 24/28 (85.7%) and 29/32 (90.6%), respectively. Actin, desmin, and S-100 were not expressed in all cases investigated (9, 10, and 6 respectively). The Ki-67 labeling index evaluated in 11 cases ranged from 30–90% (mean 57.6%). The number of tumor-associated T lymphocytes (TIL), either CD3 or CD8, reported for cases was registered as low.

As reported in Table 1, *BRAF* V600E mutation is the prominent molecular feature of CRbCs examined so far, occurring in 13/22 (60%) cases analyzed. By contrast, due to the mutual exclusivity of *KRAS* and class I *BRAF* mutations such as *BRAF* V600E, *KRAS* variants were observed at low frequency (2/17, 12%) and basically only in *BRAF* wild-type tumors. Concurrent mutations in both genes were very uncommon (1/17, 0.06% of cases).

Moreover, as in colorectal carcinomas, MSI was strongly associated with *BRAF* V600E mutation and was observed in seven out of eight *BRAF* mutant cases. However, although a significant positive association between these two markers is also confirmed in these rare tumors, the frequency of *BRAF* mutant/microsatellite stable (MSS) CRbC was unexpectedly high (6/13 cases; 46%) compared with the low incidence of this molecular subset among colorectal cancers (CRCs) [27]. Molecular features strongly associated with *BRAF* colorectal cancers such as a high frequency of CIMP and rare loss or mutation of the *TP53* gene have been scarcely studied in CRbCs (Table 1). Finally, recent next-generation target sequencing of *SMARCB1* (INI-1) and *CROCC* genes highlighted point mutations of the *CROCC* gene in 4/10 (40%) cases and no *SMARCB1* (INI-1) genetic alterations [4].

## 3. Discussion

The neoplasms reported in this study comprising those revised from the literature, displayed peculiar rhabdoid features and were associated with an ominous clinical course with a large majority of patients surviving for less than six months. The diagnostic hallmark of these neoplasms is the presence of rhabdoid cells characterized by round eosinophilic aggregates of intermediate filaments that displace the nucleus to the cell periphery. Other consistent morphologic features are: The non-cohesive growth of tumor cells, the scarcity of tumor stroma, the abundance of tumor infiltrating neutrophils, and the scarcity of lymphocytic infiltration. The main immunohistochemical findings are: The expression of vimentin and pancytokeratin within filamentous cytoplasmic inclusions, the loss of membranous E-cadherin, the nuclear dislocation of β-catenin, the lack or reduced expression of important markers of colonocyte differentiation such as CK20 and CDX2, the marked nuclear p53 accumulation, and the high proliferative Ki-67 index.

The majority of CRbCs consist solely of rhabdoid cells and are indicated as “pure”, while other CRbCs combine a rhabdoid component with an adenocarcinoma component most frequently focal and confined to the tumor periphery and are designated as “combined”. The presence of a transitional zone in combined CRbCs with a continuum between rhabdoid and non-rhabdoid cells indicates that rhabdoid cancers cells (RbCs) might have originated from dedifferentiated primary colorectal cancer [5,28].

The most relevant immunohistochemical finding for the diagnosis of CRbC is the coexpression in tumor cells of pancytokeratin and vimentin. This was found in all CRbCs but not in PDMCs examined. Coexpression of a mesenchymal marker such as vimentin and epithelial markers is one of the phenomena that characterize the process of epithelial–mesenchymal transition (EMT) [29]. This is a unique process in which cells lose epithelial features and acquire mesenchymal qualities [30]. The process of EMT is characterized by the reduction of epithelial markers and increase of mesenchymal markers [31]. E-cadherin is the most important mediator of cell adhesion in epithelial tissues and loss of E-cadherin is a crucial step in EMT. During EMT, loss of E-cadherin is associated with the release of β-catenin, which is consequently translocated to the nucleus where it activates the WNT signaling pathway [32]. In colorectal cancer, altered expression of E-cadherin and β-catenin and progressive increase of vimentin in late stages are associated significantly with aggressive tumor cell behavior and, furthermore, confer resistance to cancer drugs [33,34]. In addition, in a recent study [35], it has been demonstrated that the gene expression profile of tumor budding regions in CRC closely matches that of consensus molecular subtypes 4 (CMS4) (mesenchymal) subtype, while the bulk presents a CMS2 (epithelial profile).

Previous immunohistochemical results demonstrating loss of membranous E-cadherin in CRbC [2,3,12] and our results demonstrating β-catenin nuclear localization and loss of colonic epithelial markers such as CK20 and CDX2 support the pathogenetic involvement of EMT as an essential player in the heterogeneous make-up of CRbC. In addition, the loss of membranous E-cadherin and β-catenin suitably explains the discohesive histologic pattern of CRbC.

Cells undergoing EMT maintain the same genomic background in both mesenchymal and epithelial states, but during the progression of EMT, the gene expression profile significantly changes. A series of protein complexes, known as chromatin remodelers, are crucial to mediate this event as they can slide, destabilize, or relocate nucleosomes in an ATP-dependent manner [36]. The SWI/SNF mating-type switching (SWI) and sucrose nonfermenting (SNF) subfamily has specifically been investigated in malignant rhabdoid tumors, pediatric and highly lethal neoplasms of the kidney and brain where *SMARCB1* (INI-1) is frequently mutated either at germline or at somatic level [37]. To date, the role of *SMARCB1* (INI-1) inactivation remains to be determined in CRbC and only few studies reported SMARCB1 (INI-1) immunohistochemical loss in a small subset of CRbCs that were frequently *BRAF*-mutant, MSI, and CIMP [1,3,11]. These data allowed to hypothesize that *SMARCB1* (INI-1) may occur as a secondary molecular event during EMT in a subset of CRCs characterized by *BRAF* V600E mutation, MSI, and CIMP, virtually conferring a rhabdoid phenotype [2]. In line with this hypothesis, Wang et al. [10] demonstrated that loss of *SMARCB1* (INI-1) expression occurs at least focally in 0.46% of 3051 CRCs and is associated with higher grade, larger tumor size, poorer survival, MSI, and *BRAF* V600E mutation.

In this context, our study sheds some light on the biological features of this rare entity thanks to a genetic/epigenetic comparative analysis of CRbCs and PDMCs showing *BRAF* V600E as a common prominent genetic feature. A first important finding of our analysis was that CRbC mainly included *BRAF* mutant/MSS cancers without CIMP. By contrast, PDMC only comprised *BRAF* mutant/MSI and CIMP cancers. Two *BRAF* mutant/MSI cases were observed among CRbCs and in one case we could exclude CIMP. Interestingly, both these cases showed a reduced SMARCB1 (INI-1) expression but not a complete loss of the protein as we found in the remaining five CRbCs.

To date, due to their rarity, the *BRAF* mutant/MSS colorectal cancers have not been as thoroughly studied as *BRAF*/MSI cancers. Although both subsets derive from a serrated polyp due to the presence of the *BRAF* mutation and are clinically associated with a detrimental patient outcome, *BRAF* mutant/MSS colorectal cancers diverge from *BRAF*/MSI cancers with the development of clinicopathologically and genetically distinct aberrations [27]. Histologically, *BRAF* mutant/MSS cancers show more adverse morphological features compared with *BRAF*/MSI cancers, such as frequent tumor budding; high-grade neuroendocrine carcinomatous component; a lack of tumor infiltrating lymphocytes; frequent lymphatic, perineural, and venous invasion; and increased lymph-node metastases [38,39,40]. In addition, it is of interest to recall that in one study [41] *BRAF* mutant CRCs on the basis of gene expression have been split in two subtypes called BM1 and BM2. The subtypes displayed differences in overall survival (OS) and progression free survival (PFS). The enrichment of BM1 group in EMT signature and CMS4 consensus subtype correlates with poor survival of patients.

For the first time, with this work, we suggest that *BRAF* mutant/MSS cancers include the rare entity of CRbCs, characterized by a strong activation of EMT and complete loss or reduced expression of SMARCB1 (INI-1). Moreover, a recurrent finding of CRbCs in this study, was the abundance of tumor-infiltrating neutrophils which contribute to the formation of the tumor microenvironment. Although neutrophils were at first considered to possess defensive functions against cancerous cells, it has been demonstrated that some subtypes of neutrophils, known as tumor-associated neutrophils (TANs) possess a tumor-supporting function [42]. TANs contribute to tumor invasion and angiogenesis through production of matrix metalloproteinases, vascular endothelial growth factor (VEGF), and hepatocyte growth factor (HGF). Interestingly, intratumoral neutrophils in CRCs have been found to correlate closely with a malignant phenotype and to represent an independent factor of poor prognosis for the patients [43].

Molecularly, *BRAF* mutant/MSS cancers have multiple genetic aberrations that are representative of typical changes associated with both serrated and conventional pathways. Although they display hypermethylation events that commonly characterize all *BRAF* mutant cancers, this subset of tumor shows lower frequency of CIMP than *BRAF*/MSI cancers [44,45]. In line with this observation, CIMP was not found in CRbC in contrast to PDMC, analyzing the conventional panel of genes suggested to identified CIMP in tumors of the serrated pathways. Although this result does not preclude the presence of gene hypermethylation in CRbCs, the use of this gene panel may be useful to distinguish them from tumors of the classical serrated pathway.

Finally, *TP53* mutation has been correlated with advanced stages and with conventional pathway in CRCs. *BRAF* mutant/MSS cancers have been found to have a comparably high rate of *TP53* mutation as the *BRAF* wild-type cancers, whereas *BRAF* mutant/MSI were confirmed to have a low rate of mutation [44]. In our study, all but one tumor showed *TP53* mutation and no specific differences we observed comparing CRbCs and PDMCs. An interesting observation was that *TP53* mAFs were often higher than *BRAF* mAF in most of the tumors analyzed. These data emphasize a driver role of *TP53* in the early phases of the development of these tumors suggesting that in addition to the constitutive activation of the MAP kinase pathway through *BRAF/RAS* mutations, simultaneous upregulation of anti-apoptotic pathways may be crucial for the rapid and aggressive growth of these tumors.

Rhabdoid carcinomas seem to be resistant to conventional therapy used for gastrointestinal neoplasms (FOLFOX, FOLFIRI scheme associated with monoclonal antibody). Moreover, anthracycline based regimes generally used in sarcoma do not seem effective. The co-presence of *BRAF* and P53 mutations in CRbCs suggests the possible therapeutic role of a double block acting on BRAF and p53.

In summary, CRbCs are characterized by *BRAF* and less frequently *KRAS* mutations co-occurring with *TP53* mutations. Coexpression of pancytokeratin and vimentin, dense neutrophilic infiltration, loss/reduced expression of nuclear of SMARCB1/INI, and low frequency of CIMP are useful markers to recognize these rare aggressive tumors. Elucidation of the genetic and epigenetic landscape alterations of these tumors is crucial to hypothesize specific treatments with novel biological agents such as MAPK inhibitors and small molecules blocking p53 degradation and epigenetic drugs.

## 4. Materials and Methods

### 4.1. Histopathologic and Immunophenotypical Study

Formalin-fixed and paraffin-embedded tissue blocks from 11 colorectal carcinomas were retrieved from our routine surgical pathology files, dating back to 1984. We included seven neoplasms composed (at least 30%) of highly atypical tumor cells with abundant eosinophilic cytoplasm containing hyaline-like globular (rhabdoid) inclusions classified as CRbC. We also included four cases interpreted as poorly differentiated medullary carcinomas (PDMCs) showing areas with discohesive polymorphic cells with abundant eosinophilic cytoplasm and eccentric nuclei, mimicking rhabdoid cells. One of the CRbCs and two of the PDMCs showed a focal glandular component. A combined CRbC and PDMC was found in one case.

Tissue sections were stained with hematoxylin–eosin and periodic acid–Schiff/Alcian blue. The histopathologic revision evaluated the following features: Grade, mitotic index per 2 mm^2^, growth pattern, tumor budding, necrosis, vascular space invasion, perineural invasion, percentage of tumor stroma, intratumoral and peritumoral lymphocytic infiltration. For assessment of tumor budding, the Nakamura method [46] was used to score the case. The degree of tumor budding was categorized into two groups: Low grade (none or mild) and high grade (moderate or marked). Tumor-associated inflammation at the tumor margin was assessed according to the criteria of Kasajima et al. [47] and graded as absent (0), weak (1+), moderate (2+), or high (3+).

The quantity of intratumoral granulocytes was graded as: Absent (0), weak (1+), moderate (2+), or high (3+). The number of intratumoral CD8-positive lymphocytes (CD8-TIL) and peritumoral CD8-positive lymphocytes (CD8-PTL) was assessed using anti-CD8 antibodies and their relative number was evaluated using a Zeiss microscope (ocular ×10; objective 25 mm) over an average of 0.882 mm^2^.

Moreover, all cases were evaluated for immunohistochemical expression of pancytokeratin, CK7, CK20. EMA, β-catenin, SMARCB1 (INI-1), vimentin, Ki-67, p53, CDX2, and synaptophysin. The antibodies, protocols, and criteria for the evaluation of immunohistochemical expression are reported in Table S4. Positive and negative controls were used throughout. SMARCB1 (INI-1) staining was interpreted according to the criteria exposed by Wang et al. [10] and categorized as 1) negative staining, 2) focally negative staining, and 3) positive staining.

### 4.2. Molecular Study

#### 4.2.1. MSI and CpG Island Methylator Phenotype (CIMP) Analysis

MSI status was evaluated in accordance with previously reported protocols [48]. Methylation study was performed using methylation-sensitive multiple ligation-dependent probe amplification (MS-MLPA), that allows the simultaneous assessment of promoter methylation of multiple genes in a single experiment. SALSA MS-MLPA ME042-C1 CIMP Kit (MRC-Holland, Amsterdam, The Netherlands) was used to perform methylation analysis on eight gene promoters frequently methylated in CIMP tumors [49] (details in Table S5). In detail, the kit contains 31 MS-MLPA probes which detect the methylation status of promoter regions of *CACNA1G*, *CDKN2A*, *CRABP1*, *IGF2*, *MLH1*, *NEUROG1*, *RUNX3*, and *SOCS1* genes.

MS-MLPA reactions were performed according to the manufacturer’s instructions. The MS-MLPA products were analyzed on an ABI 310 Automatic DNA Sequencer (Applied Biosystems, Foster City, CA, USA) using GeneMapper 4.0 genotyping software (Applied Biosystems, Foster City, CA, USA). Values corresponding to peak size in base pairs (bp) and peak areas were used for further data processing by Coffalyser V8 software (MRC-Holland, Amsterdam, The Netherlands). All probes were adjusted to reference probes within each sample (intra-sample normalization). The methylation ratio (MR) was calculated by dividing each normalized peak value of the HhaI-digested sample by that of the corresponding undigested sample. Blood-derived DNA samples of three healthy individuals were used as unmethylated reference samples for inter-sample normalization.

Sensitivity and specificity of the MS-MLPA assay were determined by a titration experiment, mixing fully methylated DNA (CpGenome Universal Methylated DNA, Millipore) with unmethylated DNA (CpGenome Universal Unmethylated DNA, Millipore) in proportions of 0%, 10%, 25%, 50%, and 100%. Using three replicates for each concentration, we observed MR values between 0 and 0.16 for the probes of fully unmethylated samples, and between 0.28 and 0.47 for the probes of 10% methylated DNAs. MRs obtained in the titration experiment with the 10%-methylated DNA were used as cutoff values to determine aberrant methylation Ratio (MR) status of our probes as categorical variables (Table S3 shows the cutoff used for each MS-MLPA probe).

To classify a gene promoter as methylated, at least half of the probes had to show methylation. We considered a sample CIMP positive if it showed at least four out of eight methylated promoters.

#### 4.2.2. Targeted Sequencing Libraries and Massively Parallel Sequencing

Tumor DNA was obtained from formalin-fixed paraffin-embedded (FFPE) tissue using three representative 8 μm sections. The sections of every specimen were treated twice with Bio-Clear (Bio-optica, Milan, Italy). Neoplastic areas were manually microdissected for DNA extraction and contained at least 50% of tumor cells to minimize contamination by normal cells. DNA was extracted using the Maxwell® DNA FFPE Kit (Promega, Madison, Wisconsin, USA) and purified using an automatic nucleic acid purification system (Maxwell 16 system, Promega, Madison, Wisconsin, USA) according to the manufacturer’s protocol. Each sample was then quantified using Qubit dsDNA High-Sensitivity Assay kit (Invitrogen, Thermo Fisher Scientific, USA).

A targeted capture library was constructed according to the protocol Human Actionable Solid Tumor Mutations QIAseq DNA Panel (DHS-101Z, Qiagen, Hilden, Germany) that allows to analyze by NGS, specific exons or hot-spot mutations in 19 oncogenes (*BRAF, PDGFRA, EGFR, KRAS, NRAS, KIT, AKT1, ALK, CTNNB1, ERBB3, ESR1, FOXL2, GNA11, GNAQ, IDH1, IDH2, MET, RAF1, RET*) plus the whole exonic regions of *ERBB2, PIK3CA,* and *TP53 (*Table S6*)*. This gene panel covers a total of 15,160 bp with 170 amplicons with a mean of 150 bp. Libraries were generated starting from 40–100 ng of FFPE DNA. Genomic DNA samples were first fragmented, end repaired, and A-tailed within a single multi-enzyme reaction. Prior to target enrichment and library amplification, each original DNA molecule was assigned a unique molecular identifier (UMI) containing a 12-base random sequence. After cleanup of adapter-ligated DNA using QIAseq beads, target enrichment was performed post-UMI assignment through eight cycles of targeted PCR using one region-specific primer and one universal primer complementary to the adapter. After cleanup of target enrichment, a universal PCR and cleanup of the amplicons were ultimately carried out. Equal volumes of individuals libraries were pooled at 4 pm.

Bead emulsion for immobilization and clonal amplification were performed with the Ion OneTouch2 System (Thermo Fisher Scientific, Waltham, Massachusetts, USA) and Ion OneTouchES instruments (Thermo Fisher Scientific, Waltham, Massachusetts, USA). Barcoded libraries of 8–10 samples were sequenced on an Ion S5 XL System (A27214, Thermo Fisher Scientific, Waltham, Massachusetts, USA) according to the manufacturer’s instructions using 500 flows.

#### 4.2.3. Next-Generation Sequencing Data Analysis

Upon completion of the sequencing run, unmapped BAM (uBAM) files were imported into the CLC Genomics Workbench (Qiagen Bioinformatics version 12, Hilden, Germany). Sequencing data were analyzed using the Biomedical Genomics Analysis plugin, which allows to align reads to the reference genome (UCSC build hg19), UMI counting, read trimming, and variant identification. Data were filtered ensuring a coverage of at least 500× and an allelic fraction of 5%. In order to detect only mutations with a deleterious effect on protein functions, both synonymous mutations and variants described in the 1000 Genome Project were filtered out.

All subjects gave their informed consent for inclusion before they participated in the study. The study was conducted in accordance with the Declaration of Helsinki, and the protocol was approved by the Ethics Committee of Varese (Project identification number: 0008465).

### 4.3. Literature Review

For comparison of clinicopathologic data, prognostic parameters, and genetic profiles with those of our tumor series, we performed a thorough review of the MEDLINE literature, for colorectal carcinomas reported as rhabdoid carcinoma, carcinoma with rhabdoid features, rhabdoid tumor, malignant rhabdoid tumor, pleomorphic carcinoma, giant cell carcinoma, and undifferentiated carcinoma. We report in Table S3 all cases that we have critically reviewed and accepted as they demonstrated similar histology and immunophenotype as the cases reported in the present study.

## 5. Conclusions

CRbCs are highly aggressive tumors and currently no specific protocols are available with proven efficacy. Although they show a wide phenotypic heterogeneity and molecular complexity, our study suggests that an integrated analysis of morphological, immunohistochemical, and molecular traits helps to recognize these uncommon tumors. Specifically, co-occurrence of *BRAF* and *TP53* mutations, simultaneous expression of pancytokeratin and vimentin, dense neutrophilic infiltration, loss/reduced expression of nuclear of *SMARCB1* (INI), and low frequency of CIMP are valuable markers to identify CRbCs. Elucidation of their genetic and epigenetic landscape will be critical in guiding the clinical development of personalized therapeutic treatments.

## Figures and Tables

**Table 1:** Molecular data of colorectal rhabdoid carcinomas (CRbCs) previously reported.

Studies	MSI Status	*BRAF*	*KRAS*	*PIK3CA*	*CROCC*	*INI-1 ^§^*	*TP53*	CIMP
Kono et al. 2007 [5]	MSS	-	WT	-	-	-	-	-
Samalavicius et al. 2013 [6]	MSS	V600E	WT	-	-	-	-	-
Lee et al. 2013 (case 1) [7]	MSS	WT	WT	-	WT	WT	-	-
Lee et al. 2013 (case 2) [7]	MSS	V600E	WT	-	WT	WT	-	-
Agaimy et al. 2014 [1]	MSI	V600E	-	-	-	NEG	-	CIMP
Moussaly et al. 2015 [8]	MSI	-	-	-	-	-	-	-
Kalyan et al. 2015 [9]	MSS	WT	Q61H	WT	-	POS	R273H	-
Wang et al. 2016 (case 1) [10]	MSS	V600E	-	-	-	POS	-	-
Wang et al. 2016 (case 2) [10]	MSI	V600E	-	-	-	NEG	-	-
Wang et al. 2016 (case 3) [10]	MSS	V600E	-	-	-	POS	-	-
Agaimy et al. 2016 [11]	MSS	-	-	-	-	POS	-	-
Remo et al. 2018 * [4]	MSS	WT	WT	-	-	-	-	-
Remo et al. 2018 * [4]	MSS	WT	WT	-	-	-	-	-
Remo et al. 2018 * [4]	MSS	WT	MUT	-	-	POS	-	-
Remo et al. 2018 * [4]	MSI	V600E	-	-	-	POS	-	-
Remo et al. 2018 (RC1) [4]	MSI	V600E	WT	-	A161S	WT	-	CIMP
Remo et al. 2018 (RC2) ** [4]	MSI	V600E	-	-	V1885A	WT	-	CIMP
Remo et al. 2018 (RC5) [4]	MSS	V600E	WT	-	WT	WT	-	-
Remo et al. 2018 (RC6) [4]	MSS	V600E	G12V	-	WT	WT	-	-
Remo et al. 2018 (RC7) [4]	MSI	V600E	WT	-	WT	WT	-	-
Remo et al. 2018 (RC8) [4]	MSS	WT	WT	-	WT	WT	-	-
Remo et al. 2018 (RC9) [4]	MSI	V600E	WT	-	S1320I	WT	-	-
Remo et al. 2018 (RC10) [4]	MSS	WT	WT	-	WT	WT	-	-
Remo et al. 2018 (RC11) [4]	MSI	WT	WT	-	A1510T	WT	-	-
Remo et al. 2018 (RC12) [4]	MSS	WT	WT	-	WT	WT	-	-

Legend: (-)—result not available; MSI—microsatellite instability; MSS—absence of microsatellite instability; CIMP—CpG island methylator phenotype; INI-1 ^§^—molecular or immunohistochemical results were indicated: POS or NEG correspond to INI-1 positive expression or negative expression, respectively. WT indicates absence of gene mutation; * CRbC quoted by Remo et al. 2018 [4] as personal communication by Sanchez P.A.; ** previously published by Pancione et al. 2011 [12].

**Table 2:** Clinicopathological data of CRbCs and poorly differentiated medullary carcinomas (PDMC) included in our study.

Cases	Gender	Age	Site	Size (cm)	Type	Metastases	Stage	Treatment	Outcome *
CRbC 1	F	63	Hepatic flexure	10	Pure	N	IIIC	Surgery	2 months
CRbC 2	F	76	Sigmoid colon	4	Pure	-	-	Surgery + CT	7 months
CRbC 3	M	85	Splenic flexure	6	Pure	N, L	IVA	Surgery	2 months
CRbC 4	M	65	Cecum	6	Pure	N	IIIB	Surgery	216 months (alive)
CRbC 5	M	63	Rectum	6	Pure	N	IIIC	Surgery	10 months
CRbC 6	M	64	Hepatic flexure	6	Combined	N	IIIB	Surgery	-
CRbC 7	F	77	Ascending colon	7	Combined	absence	IIA	Surgery	187 months (alive)
PDMC 1	M	79	Ascending colon	10	-	N	IIIC	Surgery	11 months
PDMC 2	F	94	Ascending colon	8	-	N	IIIC	Surgery	5 months
PDMC 3	F	53	Sigmoid colon	13	-	N	IIIC	Surgery	-
PDMC 4	M	73	Cecum	8	-	N	IIIC	Surgery	124 months (alive)

Legend: F—female; M—male; N—lymph node; L—liver; CT—chemotherapy, (-)—data not available; * time from diagnosis to death was reported or to the last follow-up if the patient was alive.

**Table 3:** Main immunohistochemical results in CRbCs and PDMCs included in this study.

ID	Vim	Pancytokeratin	INI-1	Nuclear	p53	CD8+ (PLI/ILI)
				β-Catenin		
CRbC 1	3+	3+	0	0	3+	5/15
CRbC 2	3+	3+	0	3+	3+	10/3
CRbC 3	3+	1+	0	2+	3+	5/6
CRbC 4	2+	3+	0	0	3+	73/29
CRbC 5	3+	2+	0	3+	2+	53/13
CRbC 6	3+	3+	1+	2+	3+	61/38
CRbC 7	1+	1+	1+	2+	2+	15/16
PDMC1	0	3+	3+	3+	0	23/4
PDMC2	0	3+	3+	3+	3+	26/2
PDMC3	0	3+	3+	1+	0	54/5
PDMC4	0	n.a.	3+	3+	2+	106/15

Legend: 0—negative; 1+—(1–30%); 2+—(31–60%); 3+—(>60%); n.a.—not available; Vim—vimentin; PLI—peritumoral lymphocytic infiltrate (number per 0.882 mm^2^); ILI—intratumor lymphocytic infiltrate (number per 0.882 mm^2^).

**Table 4:** Results of MSI, CIMP, and mutation analyses in CRbC and PDMC included in this study.

ID	MSI	CIMP	*BRAF* (mAF)	*KRAS* (mAF)	*NRAS* (mAF)	*PIK3CA* (mAF)	*TP53* (mAF)
CRbC 1	MSS	no CIMP	V600E (7.5)	WT	WT	WT	R273C (11.9)
CRbC 2	MSS	no CIMP	V600E (22.6)	WT	WT	WT	R273C (29.7)
CRbC 3	MSS	no CIMP	G466A (25.1)	WT	G12D (28.2)	WT	G245S (55.7)
CRbC 4	MSS	no CIMP	WT	Q61K (19.7)	WT	WT	R273C (30.9)
CRbC 5	MSS	no CIMP	WT	G13D (86.5)	WT	WT	P278A (56.9)
CRbC 6	MSI	no CIMP	V600E (21.2)	WT	WT	H1047R (29.1)	R273C (12.3)
CRbC 7	MSI	-	V600E *	-	-	-	-
PDMC 1	MSI	CIMP-H	V600E (43.31)	WT	WT	WT	* P152fs, 18 (37.9) * V73fs, 50 (34.3)
PDMC 2	MSI	-	V600E (21.85)	WT	WT	WT	WT
PDMC 3	MSI	CIMP-H	V600E (68.89)	WT	WT	R93Q (33.6) M772I (39.5)	V272M (33.1)
PDMC 4	MSI	CIMP-H	V600E (43.27)	WT	WT	WT	Y163C (41)

Legend: (-)—data not available; mAF—mutated allelic fraction; * this mutation was found using Real-Time PCR Easy ® BRAF kit (Diatech Pharmacogenetics, Jesi, Italy). CIMP—CpG island methylator phenotype: CIMP-H—CpG island methylator phenotype at high frequency; no CIMP—CpG island methylator phenotype; absence of MSI—microsatellite instability; MSS—microsatellite stable; WT—wild-type.

## Supplementary Materials

The following are available online at https://www.mdpi.com/2072-6694/11/9/1252/s1: Table S1: Raw data obtained in CIMP analysis using SALSA MS-MLPA ME042-C1 CIMP Kit. To classify a gene promoter as methylated, at least half of the probes had to show methylation. We considered a sample CIMP positive if it showed at least four out of eight methylated promoters; Table S2: Variants passing filters observed in CRbC and in PDMC; Table S3: Reported colorectal carcinomas with rhabdoid features; Table S4: Antibodies used and immunohistochemical protocols; Table S5: List of the eight gene promoters analyzed for hypermethylation status using SALSA MS-MLPA ME042-C1 CIMP Kit; Table S6: Genes analyzed in the Human Actionable Solid Tumor Mutations QIAseq DNA Panel.
