# Supplementary material for: BRAF Mutation in Colorectal Rhabdoid and Poorly Differentiated Medullary Carcinomas
Source: Cancers (Basel). 2019 Aug 26;11(9):1252. doi: 10.3390/cancers11091252 (PMC6770689; doi:10.3390/cancers11091252)
Supplement: Supplementary file 1 [file cancers-11-01252-s001.zip › Table S4.docx]

Table S4. Antibodies Used and Immunohistochemical Protocols.

| **Primary Antibody** | **Clone** | **Working Solution** | **Treatment** | **Manufacturer** |
| --- | --- | --- | --- | --- |
| CD3 (rabbit monoclonal) | 2GV6 | Pure | MW 5 min’×2 CB, pH 6 | Ventana |
| CD8 (rabbit monoclonal) | SP57 | 1:2 | MW 5 min’×2 CB, pH 6 | Ventana |
| PD-L1 (rabbit monoclonal) | SP142 | 1:40 | MW 5 min’×4 EDTA, pH 8 | Spring |
| p53 (mouse monoclonal) | DO-7 | 1:500 | MW 5 min’×4 CB, pH 6 | Dako |
| CDX2 (mouse monoclonal) | CDX2-88 | 1:50 | MW 5 min’×4 CB, pH 6 | Biocare |
| Ki-67 (mouse monoclonal) | MIB-1 | 1:100 | MW 5 min’×4 CB, pH 6 | Dako |
| Synaptophysin (rabbit polyclonal) | Polyclonal | 1:2 | MW 5 min’×4 CB, pH 6 | Ventana |
| EMA | E29 | 1:100 | MW 5 min’×2 CB, pH 6 | Dako |
| CK AE1/AE3 | PCK26 | 1:2 | MW 5 min’×4 CB, pH 6 | Ventana |
| CK7 | SP42 | 1:2 | MW 5 min’×2 CB, pH 6 | Ventana |
| CK20 | KS20.8 | 1:100 | MW 5 min’×2 CB, pH 6 | Dako |
| b-catenin | 14 | Pure | MW 5 min’×4 CB, pH 6 | Ventana |
| SMARCB1(INI-1) | MRQ27 | Pure | MW 5 min’×6 CB, pH 6 | Ventana |
| Vimentin | V9 | Pure | MW 5 min’×2 CB, pH 6 | Ventana |

Abbreviations: CB—[citric acid](https://www.sciencedirect.com/topics/medicine-and-dentistry/citrate) [antigen retrieval](https://www.sciencedirect.com/topics/medicine-and-dentistry/antigen-retrieval) buffer; [EDTA](https://www.sciencedirect.com/topics/medicine-and-dentistry/edetic-acid)—[ethylenediaminetetraacetic acid](https://www.sciencedirect.com/topics/medicine-and-dentistry/edetic-acid); MW—microwave antigen retrieval solution.

[Immunohistochemistry](https://www.sciencedirect.com/topics/medicine-and-dentistry/immunohistochemistry) was performed manually; formalin-fixed paraffin-embedded sections were mounted on poly-l-lysine–coated slides, deparaffinized, and hydrated through graded alcohol to water. Endogenous [peroxidase activity](https://www.sciencedirect.com/topics/medicine-and-dentistry/peroxidase) was quenched in 3% [hydrogen peroxide](https://www.sciencedirect.com/topics/medicine-and-dentistry/hydrogen-peroxide) in water for 20 minutes; proteolytic treatment was performed using different antigen-retrieval solutions (CB, pH 6; or EDTA, pH 8) in a domestic 750-kW microwave oven. Primary antibodies were applied overnight at 4°C and immunostained using the avidin-biotin-peroxidase complex (ABC) method or the MACH4 system. For ABC method, the sections were incubated with biotinylated anti-mouse immunoglobulins and ABC complex, each for 1 hour at room temperature. The immunoreaction was developed with 3.3′-diaminobenzidine tetrahydrochloride as chromogen and nuclei were counterstained with [hematoxylin](https://www.sciencedirect.com/topics/medicine-and-dentistry/haematoxylin). Finally, the sections were dehydrated.
